# Effects of Caseworker Screening on Employment and Health: Quasi-Experimental Evidence From the Swedish Sickness Insurance Program

**DOI:** 10.1177/0193841X251358288

**Published:** 2025-07-09

**Authors:** Pathric Hägglund, Per Johansson, Kristian Persson

**Affiliations:** 1 Stockholm University, Stockholm, Sweden; 2 151672 Uppsala University, Uppsala, Sweden; 3 YMSC, Tsinghua University, Beijing, China; 4 The Swedish Social Insurance Inspectorate, Göteborg, Sweden

**Keywords:** absenteeism, social insurance, health, earnings, H55, J22, G22

## Abstract

This paper analyzes the effects of caseworker screening of doctors’ medical sick-leave certificates. The analysis uses data from people appealing caseworkers’ denial of their benefit claims. Caseworkers at four units made decisions on the appeals. The distribution of the cases to the four units was based on the appealing person’s birth date. One of the units was much stricter than the others (7.8% approved in contrast to 16.1% for the others). This allows us to use birth date as an instrument to estimate the effects of being denied sickness benefits. We find that the denial of sickness benefits, on average, has (i) positive effects on the labor-market outcomes and (ii) no negative effects on health outcomes. As the more liberal units deny sickness benefits for most screened medical certificates, we conclude that caseworker screening is very important in separating meritorious from non-meritorious claims. Doctors’ conflicting roles make it difficult for them to act in the best of interest of both their patients and society.

## Introduction

It is well known that substantial moral hazard can exist in the sickness and disability insurance (SI and DI) programs (see e.g., Godard et al. (2022) and Eliason et al. (2019)). The implication is that there is a need to screen eligibility of the people applying for benefits. In most countries with SI and DI programs, doctors have a gate-keeping role in the form of providing written medical certificates to caseworkers who approve or deny (cf. Liebert, 2019). However, the primary objective of physicians is to be a healer. For patients presenting with symptoms, it can be difficult for doctors to question the reported symptoms—that is, to act as gatekeepers—since doing so may seem unethical and could interfere with providing appropriate medical care.^1^

The two-sided role of treating physicians makes the role of a caseworker or an external specialist important. Evidence for this comes from Liebert (2019), who evaluates the impact of the introduction of external medical review of DI applicants in Switzerland. He finds that external medical review reduced DI inflow by 23% and increased labor-market participation of claimants. The positive effect is mainly derived from those with difficult-to-diagnose conditions. Liebert concludes that the reduction stems mainly from a reduction in type-II errors.

This paper analyzes effects on labor-market outcome and health of caseworker screening. The analysis exploits the fact that previously denied sickness benefit (SB) applicants appealing the decision in 2009–2012 were assigned to four different specialist units based on their birth date. Although following the same eligibility criteria, the units systematically differ in the level of stringency. Thus, the paper contributes to the literature on effects of screening in the SI program. However, as the Swedish SI program is intimately related to the DI program and almost all individuals on disability benefits (DB) have a history of long sick-leaves we also contribute to the literature on targeting and screening in the DI program.

Denied applications have previously been used to study targeting and classification errors in the DI literature. Bound (1989) is the classic study to analyze the earnings of rejected DI applicants. More recent contributions are French and Song (2014), Maestas et al. (2013), Low and Pistaferri (2015), and Manasi and Yue (2019).

Since assignment to different specialist units was based on birth date the two groups should be similar by design. If this is the case, then the analysis consists in comparing outcomes of those assigned to the restrictive unit to those of the three other units. The identification strategy is thus similar to that used in French and Song (2014) and Maestas et al. (2013), who analyze the effects of denial in the U.S. DI program based on differences in lenience among judges and examiners.

As we observe health of the denied person this allows us to also indirectly test the balance between Type-I and Type-II errors in awards. The idea is that, if the health- and labor-market outcomes are no worse for those assigned to the more restrictive units this provides evidence of the importance of caseworker screening in reducing Type-II errors.

As in Liebert (2019) we analyze the effect of external reviews. In the Liebert case, the external reviewer is a medical specialist examining doctor’s assessments. Thus, the effect in Liebert could be from differences in strictness but also from differences in quality of assessment. In our case, the external reviewer is a caseworker that is a specialist who is providing a second opinion—on a colleague’s denial—of a doctor’s certificate. However, as we are using variation in assessment—that can be considered as random—between specialist units, the effects we are estimating should be the effect of differences in screening strictness only.

All previous studies on increased screening strictness in the SI program that we have reviewed have found a reduction in take-up rate of sick-leave (see e.g., De Jong et al., 2011; Hartman et al., 2013; Johansson & Lindahl, 2013; Markussen et al., 2018).^2^ In addition to focusing on analyzing caseworker screening, our contribution to this literature is that we are the first paper analyzing the effects of screening on health outcomes. Additionally, we contribute to this literature by analyzing more long-term effects than previously studied.

The analysis is based on administrative data from the Social Insurance Agency (SIA) and the National Board of Health and Welfare. Labor-market outcome is measured by labor income, number of days with SB, and disability benefits (DB) take-up rates. Health is measured by number of outpatient care visits, number of inpatient care days, and drug consumption. We estimate yearly effects up to 3 years after the appeal decision.

We find that denial of SB, on average, (i) reduced number of sick-leave days, (ii) reduced risk of receiving DB within 3 years, and (iii) increased labor income. We find *no* evidence that denial negatively impacted health on average. As no negative effects on health were found, it suggests that stricter screening of appealed cases for SB reduced Type-II errors in the SI program and thus that it produces a more deserving pool of DI beneficiaries. As more liberal units deny sickness benefits for almost 84% of their screened medical certificates, we also conclude that caseworker screening in general is very important in separation of meritorious from non-meritorious claims. Doctors’ conflicting roles make it difficult for them to act in the best interest of both their patients and society.

The paper unfolds as follows. Section 2 discusses Swedish institutions. Section 3 describes the quasi-experiment and data together with a descriptive analysis. The results are presented in section 4, and section 5 concludes with a discussion.

## Institutions

This section provides a short description of the Swedish social insurance (section 2.1) and health care systems (section 2.2).

### The Social Insurance System

Both employed and unemployed individuals are covered by the public SI and DI program administrated by the Swedish SIA. Most workers are also covered by an unemployment insurance (UI) program. Unemployed individuals (regardless of whether or not they are covered by the UI program) have access to the SI program.

#### The SI Program

The employer is responsible for sick pay the first 14 days of the individual’s sick-leave (the first day is not compensated). After that, the SIA pays SB. For an unemployed individual, the SIA pays SB starting from day 2 of the individual’s sick-leave. Compensation covers a little less than 80% of foregone earnings^3^ up to a cap (USD 2,220 per month in 2012). Furthermore, collectively agreed compensation typically covers up to 90% of earnings during the first 3 months. From the fourth month onwards, total compensation is typically back at 80%, at least among highly paid workers. After a year, compensation decreases from 80% to 75% of earnings.

During the first 7 days of an illness, the individual decides if (s)he needs to be absent from work. The individual merely has to inform the employer (or the SIA if unemployed) that (s)he is ill. As of the eighth day, a medical certificate is required. The doctor provides medical evidence about applicants’ health condition related to work requirement of the individual. In the certificate, the doctor recommends length and extent of sick-leave. All doctors have the right, and are obliged, to write a medical certificate if they believe their patient requires one. There are no sanctions on doctors writing flawed certificates and they are *not* paid to write certificates by the county council (see the section below for the organization of health care).

The caseworker takes information in the medical certificate along with other factors in the application (e.g., the applicant’s occupation) and it is their duty to ultimately determine eligibility for SB—not the doctor. Thus, based on this information, the caseworker decides whether the illness has reduced the individual’s capacity to work. Most often full-time benefits are paid. However, sick-leave can also be part-time, which implies SB being paid for 75, 50, or 25% work limitations.

There are eligibility checks at three and 6 months in a sick-leave spell, stipulating increasingly higher requirements to qualify for SB. Workers are entitled to SB for 90 days if they are unable to perform their ordinary duties, or other temporary work, at their workplace. After 90 days, entitlements to SB hold only if workers are unable to perform any type of work at their work place. After 180 days, entitlement to SB holds only if the individual cannot do any type of work on the labor market.^4^ However, this rule holds *only* if the worker is unlikely to return to work with their employer before day 366. For unemployed workers applying for SB, working capacity is judged against the labor market as from day 1. Between July 2008 and January 2016, there was a maximum time limit of 2.5 years with SB, which no longer exists. A worker who had received SB for 2.5 years was disqualified from further SB for at least 3 months.

**Figure 1.**
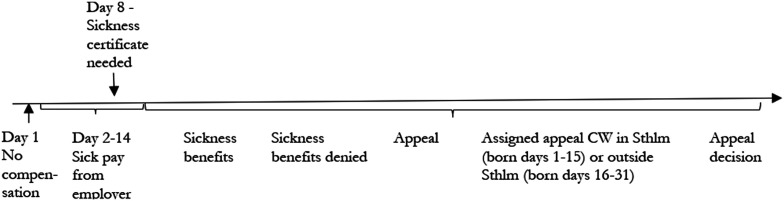
The Swedish Sick-Leave Process. “CW” is Caseworker and “Sthlm” is Stockholm

SB is denied if the caseworker does not find the individual’s capacity to work is being reduced. The applicant can then appeal.^5^ The appeal must be submitted within 60 days from the time period when (s)he was denied SB. Appeals concern both new sick-leave spells and ongoing sick-leave spells. An appeal of a denial in an ongoing sick-leave spell concerns a sickness certificate that extends and an “ongoing” spell. For new spells, the appeal concerns denied SB, that is, sick-leave spells longer than 14 days.^6^ In general, it takes around 3 months from denial of SB until a decision of the appeal is taken in the reassessment. The mean number of days to reach a decision is 93 in our data. Figure 1 sums up essentials of the sick-leave process.

The appeal assessment was conducted by specialized caseworkers in dedicated assessment units within the SIA. The caseworkers were instructed to perform a comprehensive new examination of each case. The reassessment was to be based on the same documentation as the original decision. However, if the applicant provided additional information in their appeal, further investigation might be necessary for a thorough examination.

If SB is denied in the reassessment, the applicant can appeal to administrative courts on different levels up to the supreme administrative court. These appeals are very rare and not considered in the analyses.

#### Adjoining Social Insurance Programs

Full income-related DB amounts to about 64% of previous earnings up to the same cap as in the SI scheme. The collectively agreed compensations typically cover up to 80% of earnings. Hence, going from SB to DB does not involve a drastic reduction in a compensation level. If the individual is younger than 30 years, (s)he is only granted DB for 3 years at a time. As in the SI, there is a possibility of receiving partial DB, which is benefits when a person can work for 75, 50, or 25%. As an employee with, say, 50% DB, can be on sick-leave from his or her job, this means that (s)he can, at the same time be on both SB and DB. If this person is not working any of the ordinary 50% working time, (s)he is considered as being full-time on sick leave. On the other hand, if (s)he is working 25%, (s)he is considered as being on part-time sick leave.

The payment from the UI program is based on previous income instead of foregone earnings as in the SI. The implication of this difference in basis for benefit payments is that benefits could be higher as sick listed than as unemployed.

### The Health Care System

The local county councils are the major financiers and providers of Swedish health care. There are 20 county councils (23 at the time of the quasi-experiment), and each council is obliged to provide its residents with equal access to health services and medical care. Health care is mostly financed through local taxes. Each county council sets its own patient fees, but a national ceiling limits the total amount that a patient pays during a 12-month period (out-of-pocket) to 1,100 SEK (USD 120). Thus, patient fees only account for about 3% of total revenues. Doctors—not nurses—have the right to write medical certificates. The daily fee for staying in a hospital is a maximum of 100 SEK (USD 11). There is free choice of providers but referral is required in some cases, particularly when patients seek specialized care or when they choose health care in another county. The county councils are allowed to contract private providers but the majority of health care is performed by public agents. For prescribed medicines, there is a maximum amount that a patient needs to pay of 2,200 SEK (USD 240) over a 12-month period.

## The Quasi-Experiment, Data, and Descriptive Statistics

Between 2009 and 2012, the reassessments were performed by caseworker specialists at four units. The allocation of cases across the four units was based on the day of the month that the individual was born: if the individual was born in the first half of the month, that is, between the 1st and the 15th, the reassessment was performed in Stockholm. If the individual was born in the second half of the month, that is, between the 16th and the 31st, the reassessment was performed in either Jönköping (2009–2010), Norrköping (2010–2012), or Göteborg (2011–2012).

Administrative data are gathered from the database at the SIA. The data includes information on all started sick-leave, unemployment and disability spells since 1994, as well as income, employment status and other socioeconomic variables. Data on yearly number of outpatient care visits, inpatient care visits and drug consumption are taken from registers administrated by the National Board of Health and Welfare. Given the low personal cost for a hospital visit and costs for drugs, these measurements should be relevant and valid health measurements (see e.g., O’Donnell, 2009).

We find that about two-thirds of appeals concern new sick-leave spells. These spells are most likely short as doctors very seldom issue a first medical certificate longer than 1 month. Total spell lengths of those appealing in an ongoing spell can, however, be very long (sometimes exceeding 2 years). Overall mean number of days to a decision of appeal in our data is 93 days with a standard deviation of 46 days. In the analysis the outcomes are measured after the decision from the appeal.

The allocation procedure was not followed 100%, possibly due to transfers caused by temporary peaks in workload.^7^ As a consequence, we use date of birth as an instrument in an instrumental variable analysis.

Descriptive statistics for the population with (1) an approved sick-leave spell during the study period, (2) those appealing, and (3) the study population are displayed in columns (1), (2), and (3) of Table 1, respectively. The most striking difference between the overall population in column (1) and the appealing population in column (2) is the much worse labor-market situation for the appealing population: on average (i) higher unemployment rate (51% compared to 16%), (ii) lower incomes (SEK 144,000 compared to SEK 233,600), (iii) more days on sick-leave (338 days compared to 126 days), and (iv) more days on DB (84 days compared to 25.5 days). We can also see that the fraction of males differs: 40% against 34% in appealing population.

**Table 1.** Descriptive Statistics, Mean (Standard Deviation), of Those Starting a Sick Spell in 2009–2012, Appealing Applicants, and Study Population

	All Starting a sick spell (1)	Appealing (2)	Study population (3)
Male	0.40	0.34	0.31
Age	44.9 (12.4)	47.3 (11.2)	46.3 (11.6)
Foreign born	0.15	0.26	0
Pre-secondary education	0.16	0.19	0.16
Secondary education	0.53	0.53	0.56
Post-secondary education	0.32	0.27	0.28
Receiving DB^a^	0.05	0.09	0
Unemployed^a^	0.16	0.51	0.47
Working income (previous year), SEK 1,000	233.6 (154.3)	144.0 (129.5)	161.9 (133.8)
Days on sick leave since 2005^b^	126.0 (257.4)	337.7 (423.2)	305.1 (401.4)
Days on DB since 2005^b^	25.6 (187.2)	84.0 (337.7)	96.5 (359.9)
Days registered at the PES since 2005^b^	136.6 (310.7)	183.3 (352.8)	174.6 (341.1)
Observations	1,180,053	19,119	11,557
For those with a started sick spell^c^			
Psychiatric diagnosis	0.19	0.21	0.22
Musculoskeletal diagnosis	0.22	0.43	0.42
Other diagnosis	0.59	0.36	0.36
Observations	**1,180,053**	6,135	3,331

^a^Refers to the status at sick-leave start (“All starting a sick spell”) and at the time of benefit denial (“Appealing”, “Study population”).

^b^Refers to spells started during and after 2005 and ended before benefit denial.

^c^This information is only available for registered sick spells, which means that we can only observe it for i) all starting a sick spell (Column 1), ii) those appealing a negative decision concerning an extension of sickness benefits (Column 2 and 3).

From bottom panel in the table, we can see that of all approved sick-leave spells 41% are due to psychiatric (19%) and musculoskeletal (22%) diagnoses. In the appealing population we do not observe the diagnosis as the spells were not approved. However, for the sub-population of individuals with an ongoing sick spell, we observe the diagnosis code of earlier spell. In this population, we can see that musculoskeletal diagnoses are almost twice as common as compared to the overall population.

Column (3) displays the statistics for the study population. In this population we have excluded foreign born, those currently receiving DB, and those younger than 20 and older than 64. This leaves us with a total of 11,557 individuals in our analysis sample. The reason for excluding foreign-born individuals is that the date of birth is often assigned rather than recorded for refugee immigrants to Sweden. Since certain dates (e.g., the 1st, 10th, and 15th) are disproportionately chosen, this undermines the validity of using date of birth as an instrument. However, it should be noted that the main results—presented below—remain qualitatively the same when we include immigrants and the younger and older population. As can be seen from column (3) and (2), the study population—in comparison to the appealing population—is positively selected: they have higher income, are less unemployed, are more educated, and are less on sick leave. We can also see that this group is even more dominated by females.

Descriptive statistics for applicants who were born in the first half and second half of the month are displayed in Table 2. As expected, the groups are very similar, and we find no statistically significant differences except with regard to the approval rate. In the bottom panel, we can also see that there are no statistically significant differences among the 3,331 individuals with ongoing sick-leave spells with regard to psychiatric and musculoskeletal diagnosis.^8^ The approval rate differs substantially between those born in the first half and those born in the second half of the month. Among those born in the second half, 16.1% had their initial decision reversed. For those born in the first half of the month, only 7.8% had their initial decision reversed.

**Table 2.** Descriptive Statistics, Mean (Standard Deviation) of Those Appealing (Study Population)

	1–15	16–31	*t-value*
Male	0.31	0.32	−1.15
Age	46.1 (11.7)	46.4 (11.4)	−1.60
Pre-secondary education	0.16	0.15	1.22
Secondary education	0.55	0.57	−1.55
Post-secondary education	0.28	0.28	0.72
Unemployed^a^	0.47	0.47	−0.74
Working income (previous year). SEK 1,000	163.6 (133.7)	160.2 (133.9)	1.36
Days on sick leave since 2005^b^	306.1 (404.1)	304.0 (398.7)	0.28
Days registered at the PES since 2005^b^	179.4 (347.1)	169.9 (335.1)	1.49
Hospital care visits within 1 year	2.8 (4.1)	2.9 (3.9)	−0.77
Inpatient care days within 1 year	0.3 (2.1)	0.3 (2.0)	−0.13
Drug doses a day within 1 year	2.2 (2.8)	2.2 (2.8)	−0.14
Approved (%)	7.8	16.1	−13.86
Observations	5.722	5.835	
Ongoing cases only^c^			
Psychiatric diagnosis	0.22	0.21	−0.67
Musculoskeletal diagnosis	0.40	0.43	1.30
Other diagnosis	0.37	0.36	−0.75
Observations	1.716	1.615	

^a^Refers to status at the time of the rejection decision.

^b^Refers to spells started during and after 2005 and ended before the rejection decision.

^c^The number of ongoing spells are 3,331. Thus, 8,226 (=11,557–3,331) are new spells.

The Swedish Social Inspectorate interviewed caseworkers in 2013 on the exact nature of the appeal process. Results from the interviews surprisingly showed that two distinct approaches had emerged (ISF, 2013, p. 55):

• Legal correctness approach: Caseworkers examined whether the original decision was legally sound. If not, the decision was amended.
• Fresh assessment approach: Caseworkers conducted a new assessment based on the original documentation, independent of the previous decision.

We suspect that caseworkers at the Stockholm unit largely adhered to a “legal correctness” approach. Cases with minor documentation deficiencies were generally not deemed relevant. Instead, attention was focused on cases where the documentation was clearly flawed or insufficient as a basis for changing the decision. Caseworkers at the other units, on the other hand, seemed to have adopted the fresh assessment approach.

## Empirical Strategy and Results

The main contribution to the screening effects literature is the simultaneous analysis of both health and labor-market outcomes. This dual focus inherently introduces a multi-significance problem.

With just two effect estimates this is easy to handle using a Bonferroni correction. However, with many observable variables measured over 3 years, we struggled with how to handle the multi-significance problem. The most usual way is that of having a main analysis to establish a few effects using Bonferroni corrected significance level. Subsequently, exploratory analyses can be carried out to investigate other potentially interesting effects without strict correction.

The problem we faced was that we could not determine which main analysis was most relevant. The core issue was how to measure an effect on health, and here we provide our rationale for not applying multiple comparison adjustments in the main analysis.^9^

It was not obvious to us how a valid measurement of health could be constructed using the available registers at the National Board of Health and Welfare. For this reason, we decided to use three health measures: hospital care visits, inpatient care visits and drug doses. However, we also wanted to estimate separate effects for the 3 years. The argument for using the three variables is that we believe they measure the same thing (i.e., if there is a positive/negative effect on health the three effects should all be non-negative/non-positive). It is not obvious when the effect should be measured. However, based on Grossman (1972) we expect any long-term effect to be larger than any short-term effect. Thus, if we have positive/negative effects after 1 year we expect any of these effects to increase with time.

Also, with the analysis on labor-market outcomes it is reasonable to believe that we could learn more by using more than one outcome measure at one time period. It is also highly plausible that the consequence of being denied benefits differs with respect to duration of sick-leave and for this reason we would like to analyze the effects separately for those in *new* and *ongoing* spells, respectively.

All in all, we are interested in estimating 36 treatment effects. The implication is that we cannot interpret single statistically significant estimates as being evidence of an effect. It is a consistent pattern of effect sizes that provides support for an effect of the stricter screening on labor market and health. For instance, we expect (i) the short-term effect on sick leave would be larger than the long-term effect and (ii) the short-term effect on DB to be smaller than the long-term effect. Furthermore, if an effect on health exists, we would expect (i) a consistent pattern across the three outcomes, and (ii) the long-term effect to exceed the short-term effect. Finally, we expect the effects in general to be larger for the population with ongoing sick-leave spells than for the population with new sick-leave spells.

Labor income is defined as yearly labor income observed in each of the 3 years following the decision. The other outcomes are measured over a year from the date of the appeal decision. We measure SB as number of days with SB (sick-leave days) in this period.^10^ DB is measured using an indicator of receiving any DB in this period. The health outcomes are measured—also in this period—as number of hospital care visits, inpatient care days, and daily drug doses.

The model used in the analysis of the yearly effects (*δ*_*t*_, *t* = 1, 2, 3) is the following:

(1) $$\begin{array}{ccc} y_{it} & = & \gamma_{t}+\delta_{t}\times Denied_{i}+\varepsilon_{it},t=1,\ldots ,3 \end{array}$$

(2) $$\begin{array}{ccc} Denied_{i} & = & \beta_{0}+\beta_{1}\times Early_{i}+\omega_{i} \end{array}$$

Here *y* is any of the six outcomes discussed above, *Denied* takes on a value of 1 if the individual is denied in the reassessment and 0 otherwise, *Early* takes on a value of 1 if the individual was born in days 1–15 of the month, and 0 otherwise and *ω*_*i*_ and *ɛ*_*it*_ are error terms, where the latter is assumed to be independent with *Early*.^11^ The other Greek letters are parameters to be estimated.

Parameters in model (1) are estimated using the two-stage least squares (2SLS) estimator. Inference is based on robust standard errors, using a robust covariance matrix estimator.

The results are presented in Table 3. From panel one and column (1), we can see that those born in the first half of the month had an 8.3% point higher denial rate than those born in the second half. For individuals on new and ongoing spells, the corresponding difference in denial rate, presented in columns (3) and (5), are 9.1 and 6.5% points, respectively. Birth date also serves as a relevant instrument for denial. It is highly significant overall and for both types of appeals (F-values are 193.27 (overall), 159.29 (new), and 45.27 (ongoing)).

**Table 3.** Estimates (est.) and Standard Errors (se) of the Effects of Being Denied Sick Leave on Labor-Market Outcomes for all Individuals, and Subdivided by New and Ongoing Sick-Leave Spells

	All		New		Ongoing	
	(1) Est./se	(2) Mean^a^	(3) Est./se	(4) Mean^a^	(5) Est./se	(6) Mean^a^
First stage	0.083***	0.161	0.091***	0.179	0.065***	0.119
	(0.006)		(0.007)		(0.010)	
Sick-leave days						
Year 1	−51.24**	61.42	−2.69	54.30	−207.06***	78.55
	(23.91)		(25.57)		(64.13)	
Year 2	−59.85**	62.40	−29.13	56.24	−153.75**	77.20
	(25.27)		(26.49)		(67.50)	
Year 3	−29.78	55.10	−38.59	51.06	11.72	64.82
	(23.33)		(24.32)		(61.58)	
1 if DB 0 else						
Year 1	−0.05	0.03	−0.05	0.02	−0.04	0.03
	(0.03)		(0.03)		(0.09)	
Year 2	−0.09*	0.06	−0.07	0.05	−0.17	0.08
	(0.05)		(0.05)		(0.14)	
Year 3	−0.15**	0.10	−0.09	0.08	−0.32*	0.13
	(0.07)		(0.07)		(0.18)	
Labor income (SEK 1000)						
Year 1	62.58**	141.24	41.19	148.99	119.59*	122.64
	(29.75)		(32.79)		(65.94)	
Year 2	56.58*	149.28	26.10	157.01	146.35**	130.74
	(32.40)		(35.52)		(74.59)	
Year 3	56.74*	154.06	31.62	163.23	124.18	132.03
	(33.88)		(36.91)		(78.19)	
Number of observations		11,557		8,226		3,331

*Note.* Estimated standard errors are corrected for the estimated instrument and using a robust covariance matrix.

*, **, and *** denote statistical significance at the 10, 5, and 1% level, respectively.

^a^Mean of dependent variable in the control group.

From column (1), we can see that denial of SB reduces future SB (panel 2) and DB usage (panel 3) and increases labor income (panel 4).

The effect on SB is statistically significant (at the 5% level) in the first and the second year while the effect on DB is statistically significant in the third year. Given the time limit in the SI of 2.5 years, together with the possibilities to enter permanent DB, the non-significant effect in the third year on SB together with the significant effect on DB is expected. Labor income is increased by an average of 62,580 SEK (USD 9,605), 56,580 SEK (USD 8,684), and 56,740 SEK (USD 8,708) in the first, second, and third year, respectively. The magnitudes of these increases in labor income are 44% (=62,580/141,244*100), 37% (=56,580/149,285*100) and 37% (=56,740/154,055*100), respectively. The effect is statistically significant the first year and also weakly statistically significant the second and third year.

The analysis supports the hypothesis that individuals who were denied in their appeal were more likely to return to work than their counterparts who were approved in their appeal.

Column (3) and (5) present the corresponding results for *new* and *ongoing* cases. The pattern is consistent with the overall results, showing negative effects on SB (panel 2) and DB (panel 3) and positive effects on labor income (panel 4). However, ongoing cases are driving overall effects on especially SB and labor income. This result may not be that surprising. It is reasonable to believe that being denied benefits in an ongoing sickness spell would be a considerably larger change in circumstances for the person compared to a denial in a new sick-leave.

Turning to effects on health, the results are presented in Table 4. We find no consistent pattern in the direction of the effects. Estimated standard errors are also large in comparison with the estimates. Hence, we cannot, based on a single estimate, refute either negative or positive health effects on average in a single year. However, given that the effects are estimated to be negative in some years and positive in other years, we believe it is fair to rule out large negative health effects on average.

**Table 4.** Estimates (est.) and Standard Errors (se) of the Effects of Being Denied Sick Leave on Health Outcomes for All Individuals, and Subdivided by New and Ongoing Sick-Leave Spells

	All		New		Ongoing	
	(1) Est./se	(2) Mean^a^	(3) Est./se	(4) Mean^a^	(5) Est./se	(6) Mean^a^
First stage	0.083***	0.161	0.091***	0.179	0.065***	0.119
	(0.006)		(0.007)		(0.010)	
Number of hospital care visits						
Year 1	−0.48	2.47	0.09	2.28	−2.08	2.93
	(0.90)		(0.95)		(2.21)	
Year 2	0.53	2.25	1.52	2.07	−2.62	2.69
	(0.93)		(1.01)		(2.25)	
Year 3	0.27	2.19	0.87	2.08	−1.67	2.46
	(0.83)		(0.89)		(2.06)	
Inpatient care days						
Year 1	0.04	0.90	−0.90	0.87	3.45	0.98
	(1.00)		(1.04)		(2.70)	
Year 2	1.46	0.91	0.23	0.89	5.85	0.97
	(1.57)		(1.53)		(4.61)	
Year 3	−0.58	1.01	−0.03	0.89	−2.23	1.32
	(1.54)		(1.55)		(4.15)	
Daily drug doses						
Year 1	−0.35	2.20	0.56	1.99	−3.14*	2.69
	(0.65)		(0.67)		(1.76)	
Year 2	−0.42	2.27	0.35	2.06	−2.71	2.76
	(0.68)		(0.70)		(1.82)	
Year 3	0.21	2.34	0.82	2.17	−1.59	2.75
	(0.73)		(0.77)		(1.83)	
Number of observations		11,557		8,226		3,331

*Note.* Estimated standard errors are corrected for the estimated instrument and using a robust covariance matrix.

*, **, and *** denote statistical significance at the 10, 5, and 1% level, respectively.

^a^Mean of dependent variable in the control group.

As shown in Table 1, the study population differs from the overall population. A relevant question, then, is whether the results presented in Table 3 would still hold if we applied weights based on observable characteristics of the broader population on sick leave at that time. To address this, we used predicted values from an estimated probability of being denied sickness benefits in the full population as weights.^12^ Results from this weighted analysis, presented in Table A2 in the Appendix, show lower precision but confirm that the inferences from Table 3 remain valid for the larger population. The results from this analysis shows lower precision but that the inference from Table 3 holds to this larger population (see Table A2 in the Appendix).

Thus, from this analysis we conclude that the behavioral effects are not explained by differences in observable characterizes in the two populations.

### How to Interpret the Effect and Who are the Compliers?

Around 95% of all SB applications are approved. We can reasonably assume that the population of appealing individuals consists of individuals with problems that are more difficult to verify than in the overall population. Caseworkers screening the appeal follow strict guidelines, but they are allowed some discretion. Screening of the appeal follows the same guidelines as the initial assessment. Additionally, unless new information has been added to the case, the screening is based on the same documentation as the original application. The main difference is that the appeal is handled by another caseworker. It is unlikely that caseworkers consider any potential individual effect (positive or negative) in their decision. If this assumption is correct, it means that we are estimating the average treatment effects for a population with a working capacity determined by differences in discretion in screening by the less strict units and the stricter unit.

To obtain an understanding of this population, we separately analyze caseworker compliance for different subgroups. From Table 5, we see that the degree of compliance is high in all groups but that some across-group differences exist. For instance, there is a difference between men (6.2% points) and women (9.3% points), and also across educational level, that is, less than high school (6.0% points), high school (7.8% points), and post high school (10.5% points). Also, compliance rate for psychiatric diagnoses, musculoskeletal diagnoses, and other diagnoses are 9.5, 4.8, and 6.8% points, respectively.^13^

**Table 5.** Share (in %) Who are Approved in the Reassessment, Overall, and Subgroups

	All	16–31	1–15*	Difference (compliers)	t-value
Overall	12.0	16.1	7.8	8.3	13.9
Men	12.3	15.3	9.2	6.2	5.7
Women	11.9	16.5	7.2	9.3	12.9
Unemployed^a^	9.0	12.2	5.7	6.5	8.5
Not unemployed	14.7	19.7	9.7	10.0	11.1
Less than high scool	10.4	13.4	7.4	6.0	4.2
High school	11.3	15.1	7.3	7.8	10.0
Post high school	14.4	19.6	9.1	10.5	8.6
Working income (previous year), SEK 1,000, above median	14.1	19.1	9.2	9.9	10.9
Working income (previous year), SEK 1,000, below median	9.9	13.3	6.3	6.9	8.9
Days on sick leave since 2005, above median^b^	8.8	12.0	5.4	6.6	8.9
Days on sick leave since 2005, below median^b^	15.2	20.2	10.1	10.1	10.8
Days registered at the PES since 2005, above median^b^	11.3	15.8	6.7	9.1	9.4
Days registered at the PES since 2005, below median^b^	12.0	16.1	7.8	8.3	13.9
Number of observations		5,722	5,835		
Ongoing sick leave					
Psyciatric diagnosis	10.0	14.7	5.3	9.5	4.3
Muskuloskeletal diagnosis	7.3	9.6	4.7	4.8	3.5
Other diagnosis	9.8	13.1	6.3	6.8	4.0
Number of observations	11,557	1,716	1,615		

^a^Refers to status at the time of the rejection decision.

^b^Refers to spells started during and after 2005 and ended before the last registered SB application.

The high compliance rate among those with a psychiatric diagnosis is no surprise. Compared to other diagnoses, psychiatric diagnoses are to a greater extent based on symptoms rather than on objective medical findings. This idiosyncrasy suggests that caseworker discretion is greater for psychiatric diagnoses. Some of the male-female difference in compliance rate is likely a direct consequence of females having a higher rate of psychiatric diagnosis (25% compared to 17%).

In the next section—focusing on analysis of heterogeneous effects—we also study differences in compliance for those classified as having good versus poor health (or good vs. bad working capacity), and those with stronger versus weaker labor-market attachment.

### Heterogeneous Effects

In this section we estimate effects separately by type of labor-market attachment, health (or working capacity) and by men and women at the time of the appeal.

As potential measurements for labor-market attachment we have education and employment status (employed or not).^14^ As potential measures of health we have three previous health measures, but measured before the decision. By combining the indicators of both latent variables into a score our hope is to use this to classify individuals into groups with good or bad labor-market attachment and good or bad health. To this end, we use principal component analysis to create these two scores.^15^

Labor-market attachment index is created by using first principal component (explaining 60% of the variation). Employment status and education have the same weight in the score. The health index is created from the first principal component (explaining 39% of the variation) of three health indicators measured during the last 6 months up to the decision of the appeal. For this score number of hospital care visits is the most important (42%) and number of inpatient care days are the least important (17%). We classify individuals with values of the score above the median as having worse labor-market attachment and health than individuals with values below the median.

The same analysis as above is conducted by each of the two strata separately. We use the findings from the main analysis to restrict the analyses to a 2 year follow-up period in all cases except the DB, where a 3 year follow-up period is used.

Results from the analyses are displayed in Table 6. From the first row of the table, we can see the relevance of the birth timing instrument for these strata. The fraction of compliers is as expected higher among those with good health than those with bad health, among those with a relatively good compared to relatively bad labor-market attachment, and among women compared to men.^16^

**Table 6.** Results from 2SLS Estimations. Stratification on Sex, Health and Labor-Market Status, and Direct Effects

	Health		Labor-market attachment		Sex	
	Good (1)	Poor (2)	Strong (3)	Weak (4)	Men (5)	Women (6)
First stage	0.085*** (0.008) [0.151]	0.081*** (0.009) [0.171]	0.098*** (0.009) [0.192]	0.066*** (0.008) [0.124]	0.062*** (0.011) [0.153]	0.093*** (0.007) [0.165]
Sick-leave days, up to 2 years	-46.57 (54.13) [91.88]	-176.12** (72.10) [155.75]	-81.38* (47.82) [109.21]	-153.83* (90.82) [141.47]	-275.19** (110.49) [116.74]	-63.40 (49.26) [127.16]
Disability benefits, year 3	-0.10 (0.08) [0.07]	-0.19* (0.10) [0.12]	-0.02 (0.06) [0.05]	-0.43*** (0.15) [0.15]	-0.009 (0.150) [0.09]	-0.191*** (0.072) [0.10]
Labor income, up to 2 years (SEK 1000)	108,98 (80,51) [313.36]	127,77 (85,17) [267.72]	24.09 (68.76) [384,61]	227.51*** (85.72) [176.98]	445.56** (182.09) [278.84]	18.61 (57.86) [296.02]
Hospital care visits, up to 2 years	0.75 (1.32) [2.76]	-0.50 (2.79) [6.68]	1.91 (1.73) [4.47]	-3.26 (3.15) [5.03]	-4.20 (4.28) [4.70]	1.32 (1.61) [4.73]
Inpatient care days, up to 2 years	0.58 .43) [0.93]	2.56 (4.05) [2.70]	2.20 (2.36) [1.65]	0.29 (4.09) [2.02]	4.06 (6.73) [2.21]	0.83 (1.86) [1.63]
Daily drug doses, up to 2 years	-0.53 (0.50) [1.17]	-0.13 (1.08) [3.29]	0.88 (0.64) [1.93]	-2.52* (1.35) [2.60]	-1.01 (1.69) [2.36]	-0.17 (0.64) [2.17]
Number of observations	5,787	5,770	6,374	5,183	3,635	7,922

*Note.* We use a robust covariance matrix to correct for estimated standard errors, shown in parentheses. Mean of the dependent variable in the control group within [ ] brackets. *, **, and *** denote statistical significance at the 10, 5, and 1% level, respectively.

Columns (1) and (2) present the results for those with good and poor health, respectively. We can see that reduction in SB days and DB take-up are more pronounced for those with relatively poor health. For those with relatively good health, effects on SB and DB are not statistically significant. For both groups, effects on income are positive and larger for the poor health group. However, none of the estimates is statistically significant. The non-negative effect on income for those with relatively poor health is interesting per se. In combination with negative effects on SB and DB, it suggests that individuals in this group are not leaving the labor force. Finally, health estimates are imprecise and none of the differences are statistically significant.

Columns (3) and (4) present the results for those with a strong and weak (i.e., more likely to be unemployed and low educated) labor-market attachment, respectively. We can see the effects on the labor-market outcomes are much more pronounced for those with weak labor-market attachment. For this group we see a reduction of 154 days of SB, a 43% point reduction in DB and an increase in labor income by 227,510 SEK (USD 34,917) (129%). Conversely, for those with relatively strong labor-market attachment we only find a statistically significant effect on SB. The effect on DB and labor income is insignificant (neither statistically nor economically). Interestingly, the signs of the coefficients for two of the health indicators suggest improved health (i.e., negative effects) from the denial for those with weak attachment but a health deterioration for those with good attachment. The differences in estimates are, however, not statistically significant.

With regard to sex differences, results shown for men and women in columns (5) and (6) are somewhat mixed. Men who are denied benefits reduce their sick-leave and increase their labor income by an average of 445,000 SEK (USD 68,298) (160%). Women on average reduce the probability of receiving DB but do not significantly increase their labor income. Finally, the health estimates are imprecise and none of the differences are statistically significant.

Our interpretation of the results regarding health and labor-market attachment is that consequences of a denial likely depend on individuals’ outside options. Those with favorable outside options—such as relatively good health and strong labor-market attachment—tend to return to work regardless of whether their application was approved or denied.

In contrast, for individuals with limited outside options, denial appears to have positive long-term effects. It may prevent people who are capable of working from becoming locked into long-term sickness insurance (SI) or disability insurance (DI) programs. Interestingly, we also observe positive health effects among individuals with weak labor-market attachment. This suggests that remaining connected to the labor market may have beneficial effects on health.

## Discussion

This paper analyzes the effects on labor-market outcomes and health of caseworker screening of doctors’ medical certificates approving sick-leave. We find that benefit denial on average reduces sick-leave, increases labor income in following years, and reduces the risk of receiving disability benefits (DB) within 3 years. We also find that the denial has no impact on health on average. Thus, the results show the importance of caseworker screening in reducing the conflicting role of doctors. These conflicts should especially be prevalent for patients with mild or moderate mental illness and/or musculoskeletal disorders, which also make up the majority of the population in this study.

The results are consistent with earlier studies showing that stricter screening in both the sickness insurance (SI) and disability insurance (DI) process can be an efficient policy to increase work resumption and decrease DI take-up rates (cf. Bound, 1989; French & Song, 2014; Low & Pistaferri, 2015; Maestas et al., 2013).

French and Song (2014) and Maestas et al. (2013), who used similar identification strategies in the U.S. DI program, find effects on labor supply that are similar to our findings on labor income. French and Song (2014) use the differences in leniency of administrative law judges in the second stage of an appeal process and conclude that a denial would have increased the labor supply by 26%. Maestas et al. (2013), on the other hand, make use of differences in examiners’ allowances rates as an instrument for denial. They find a 28% increase in labor supply in a group of denied applicants who are on the margin of being approved (comprising 23% of the population). The effect on labor supply is found to be stronger for individuals with less severe impairments. We find an effect to be around a 40% increase on labor income. We, however, for completeness, used a measure of labor supply in an extended analysis (see Table A4 in the Appendix). As no employment indicator is available in the register, we define employment using the labor income data. We follow Johansson et al. (2016, 2017) who defined a person being retired at year *t* if he or she has no income above one price base amount (PBA) at least in the 2 years after year *t*^17^ Here we use the same—but inverse—definition of being employed after the appeal decision. We find an overall effect of 31% in the first year after the denial and 20% in the second and third year. The effects in year one and years two and three are statistically significant at the (at least) 5 and 10% risk, respectively. As with income, the effect is substantially larger for individuals in ongoing sick-leave spells.

The study closest to ours is Liebert (2019), who concluded that external medical reviews in the DI process reduced DI inflow and classification errors due to incorrect awards (Type-II errors). Our results show that caseworker screening plays a similarly important role in separating meritorious from non-meritorious claims and that the screening on average improves welfare for the individuals. Furthermore, the results show that stricter screening of sick-leave spells reduces DB take-up. To this extent, the results correspond to findings in De Jong et al. (2011), who found that intensified screening in the SI program in two Dutch regions decreased long-term sickness absence and DI application rates. The results on all outcomes are also similar to results in a recent study by Ahammer and Packham (2023), who—using male workers aged 55–62—analyzed increased strictness for elderly in the Austrian DI program in 2013. They found a reduction in DB take-up rate, that the workers are more likely to remain in the labor force and *no* differences on their health outcomes.

Replacement rates in Swedish SI and DI programs are by no means extreme (Scruggs, 2006), and at the time of the study, the level of sick-leave in Sweden was also close to other comparable European countries. This suggests that the level of screening stringency was similar and that many other countries should face the same problems as Sweden with regards to monitoring and screening in social insurance programs. Autor and Duggan (2006) conclude that one important reason for the increase in DB claims after 1984 in the U.S. DI program is the extension of benefit eligibility to difficult-to-verify impairments like back pain and depression. From a policy perspective, this study shows clearly that caseworker screening of the medical certificates for sickness benefit claims are important in reducing the number of DB claims and that this is also beneficial for individuals on average.

## Supplemental Material

Supplemental Material - Effects of Caseworker Screening on Employment and Health: Quasi-Experimental Evidence From the Swedish Sickness Insurance Program Supplemental Material for Effects of Caseworker Screening on Employment and Health: Quasi-Experimental Evidence From the Swedish Sickness Insurance Program by Pathric Hägglund, Per Johansson, and Kristian Persson in Evaluation Review.

## Notes

1. See Carlsen et al. (2020) and Englund et al. (2000) on this conflict of the doctors.

2. Hartman et al. (2013) studied the effect on sick-leave of postponing the requirement of showing a doctor medical certificate from day 7 to day 14 in a sickness spell and Johansson and Lindahl (2013) analyzed effects on sick-leave of a mandatory dialogue meeting with caseworkers. Markussen et al. (2018) evaluates the effects of a compulsory dialogue meeting for long-term sick-listed workers in Norway. Overall, the meetings reduced long-term absence and the inflow to DB. De Jong et al. (2011) analyzed a cluster randomized experiment in the Dutch SI program. Results indicate that intensified screening decreased long-term sickness absence and DI application rates.

3. Forgone earnings are formally denoted SB qualifying income (SGI). SGI is the income based on estimated annual earnings. When an individual no longer has income from work, the main rule is that the SGI no longer applies. There are situations where those who are not working can keep their SGI. This is called SGI protection and applies, for example, in the event of unemployment, provided the individual is registered as a job seeker with the Public Employment Service and is actively searching for work.

4. The labor market is defined as all types of jobs in Sweden.

5. In the data we have no information on whether the appeal pertains to full-time or part-time sick-leave.

6. The reason for this is that the employer is responsible for the compensation during the first 14 days of the sick spell, so-called sick pay.

7. In our data we find that among those born in the first half of the month, 90.3% were administered in Stockholm, in accordance with the instructions. The rest were administered elsewhere. Among those born in the second half of the month, 99.6% were administered outside Stockholm, according to the rule.

8. We have also examined balance across the 23 county councils (see Table A1 in the Appendix). We found two statistically significant differences in birth dates at the 5% level for all spells. For the new and ongoing sick-leave spells we find no statistically significant differences. Thus, we do not consider the two statistically significant effects as being alarming for the validity of birth date as an instrument.

9. The approach is to some extent influenced by critical voices against hypothesis test as it leads to *p*-hacking and forking in the empirical literature. As there is no real decision to be made in science there is a valid argument for just presenting confidence intervals (see e.g., Amrhein et al. (2019) and Wasserstein et al. (2019)) and then to discuss the validity of the findings in terms of effects and no effects.

10. One sick-leave day implies full-time absence from work. Thus, a temporary sick leave of 50% during 100 days amounts to 50 sick-leave days.

11. We thus assume that *Early*_*i*_ is excluded in the “structural” model (1). This is an assumption that is impossible to verify. However, we have no prior information that birth dates should be correlated to any of the six outcomes. Furthermore, as shown in Table 2, the pre-appeal covariates are balanced between the two birth date groups, which further strengthens the validity of the assumption.

12. We estimated logistic regression models using the population described in Table 1—that is, individuals who began a period of illness during the same timeframe as those analyzed, and who were not denied benefits, were used as the comparison group in estimating the weights. All variables listed in Table 1 were included in the estimation, except for diagnosis codes and unemployment status, as these were not available for all individuals. Note that, since health outcomes are only available for the denied population, we could not apply the same weighted regression approach to the overall population for the analysis presented in Table 4.

13. All three effects are statistically significant different from zero. However, none of three differences are—on any relevant significance level—statistically significant.

14. We have no information on whether or not the person was employed when (s)he was denied benefits. We assume that all, not registered as unemployed, were employed.

15. The results from the principal component analysis are presented in Table A3 in the Appendix.

16. None of these differences is statistically significant. However, as a pattern this is interesting as all estimates are in the expected direction.

17. The Swedish PBA is regulated by law and used for various types of benefits, such as insurance payments. The PBA tracks inflation and was 42,800 SEK (about USD 6,600) in 2011.
