# Supplementary material for: Effects of Caseworker Screening on Employment and Health: Quasi-Experimental Evidence From the Swedish Sickness Insurance Program
Source: Eval Rev. 2025 Jul 9;50(1):30–54. doi: 10.1177/0193841X251358288 (PMC12715019; doi:10.1177/0193841X251358288)
Supplement: Supplemental Material - Effects of Caseworker Screening on Employment and Health: Quasi-Experimental Evidence From the Swedish Sickness Insurance Program [file sj-pdf-1-erx-10.1177_0193841X251358288.pdf]

Appendix

Table A1. Results (estimates, standard errors in parenthesis an t-test) from a linear probability regression model.

	All	t-value	*New*	*t-value*	*Ongoing*	*t-value*
County						
Stockholm	-0.10	-1.67	-0.02	-0.33	-0.01	-0.07
	(0.06)		(0.06)		(0.08)	
Uppsala	-0.08	-1.20	0.01	0.20	-0.01	-0.01
	(0.06)		(0.06)		(0.09)	
Södermanland	-0.04	-0.57	0.05	0.78	0.03	0.28
	(0,07)		(0.07)		(0.10)	
Östergötland	-0.04	-0.65	0.06	0.92	0.01	0.12
	(0.06)		(0.06)		(0.09)	
Jönköping	-0.10	-1.52	-0.01	-0.17	-0.01	-0.11
	(0.06)		(0.06)		(0.08)	
Kronoberg	-0.19	-2.75	-0.11	-1.51	-0.11	-1.03
	(0.07)		(0.07)		(0.10)	
Kalmar	-0.05	-0.75	0.05	0.69	0.00	0.04
	(0.07)		(0.07)		(0.09)	
Gotland	-0.08	-1.08	-	-1.22	0.02	0.20
	(0.07)				(0.10)	
Blekinge	-0.16	-2.42	-0.09	-0.66	-0.6	-0.67
	(0.07)		(0.07)		(0.09)	
Skåne	-0.12	-1.90	-0.04	0.37	-0.01	-0.11
	(0.06)		(0.06)		(0.08)	
Halland	-0.07	-1.03	0.02	-0.52	0.01	0.07
	(0.06)		(0.06)		(0.09)	
Västra Götaland	-0.11	-1.77	-0.03	-0.14	0.00	0.02
	(0.06)		(0.06)		(0.08)	
Värmland	-0.10	-1.58	-0.01	-0.23	-0.03	-0.33
	(0.06)		(0.06)		(0.09)	
Örebro	-0.07	-1.13	-0.02	0.52	0.08	0.90
	(0.06)		(0.07)		(0.08)	
Västmanland	-0.06	-0.96	0.03	0.34	-0.02	-0.20
	(0.06)		(0.06)		(0.08)	
Dalarna	-0.07	-1.09	0.02	-0.64	0.00	0.03
	(0.06)		(0.06)		(0.09)	
Gävleborg	-0.11	-1.73	-0.04	-0.23	0.01	0.17
	(0.06)		(0.06)		(0.08)	
Västernorrland	-0.11	-1.65	-0.01	-0.28	-0.05	-0.58
	(0.06)		(0.06)		(0.09)	
Jämtland	-0.10	-1.43	-0.02	-0.15	-	-0.54
	(0.07)		(0.07)			
Västerbotten	-0.11	-1.71	-0.01	-0.60	-0.04	0.30
	(0.06)		(0.06)		(0.08)	
Norrbotten	-0.10	-1.58	-0.04	0.85	0.03	0.80
	(0.06)		(0.07)		(0.08)	
Missing	-		0.07		0.13	
			(0.09)		(0.16)	

. Note: The number of observations for each group are as follows: all: 11,557, New: 8,226, Ongoing: 3,331.

Table A2. Estimates (est.) and standard errors (se) of the effects of being denied sick leave on labor market outcomes for all individuals, and subdivided by New and Ongoing sick-leave spells. Probability weighted OLS estimator.

	All		New		Ongoing	
	(1) Est./se	(2) Mean[a]	(3) Est./se	(4) Mean[a]	(5) Est./se	(6) Mean[a]
First stage	0.103***	0.181	0.119***	0.205	0.064***	0.118
	(0.018)		(0.025)		(0.010)	
			Sick-leave days			
Year 1	-43.17	61.20	-11.47	54.45	-188.38***	72.40
	(31.33)		(30.56)		(65.63)	
Year 2	-67.32*	63.49	-46.10	57.94	-163.00**	72.46
	(38.55)		(40.74)		(69.77)	
Year 3	-42.21	56.14	-52.69	52.75	11.14	65.08
	(33.02)		(38.59)		(62.63)	
			1 if DB 0 else			
Year 1	-0.03	0.02	-0.03	0.02	-0.04	0.03
	(0.03)		(0.03)		(0.09)	
Year 2	-0.05	0.06	-0.03	0.05	-0.16	0.07
	(0.05)		(0.04)		(0.14)	
Year 3	-0.09	0.09	-0.03	0.08	-0.34*	0.12
	(0.06)		(0.06)		(0.18)	
			Labor income (SEK 1000)			
Year 1	44.92	142.00	29.80	149.33	110.59*	126.90
	(29.25)		(30.63)		(66.58)	
Year 2	67.84**	146.72	52.17	152.71	136.69*	135.85
	(34.14)		(40.91)		(75.27)	
Year 3	74.03**	151.07	65.51	158.26	107.76	136.35
	(35.69)		(41.92)		(78.06)	
Number of observations		11,557		8,226		3,331

Note: The predicted values from an estimated probability to be denied sickness benefits is used as weights. To the end a logistic regression model is being used on the the population described in Table 1. The following covariates are used in the estimation of the probability: Male, Age, Foreign born, Pre-secondary education, Secondary education, Receiving DB Working income (previous year), Days on sick leave since 200, Days on DB since 2005, Days registered at the PES since 2005. Estimated standard errors are corrected for the estimated instrument and using a robust covariance matrix. [a]: Mean of dependent variable in the control group. *, ** and *** denote statistical significance at the ten, five and one percent level, respectively.

Table A3: Results from the principal component analysis based on the correlation matrix.

Health		
Component	Eigenvalue	Proportion explained (%)
1	1.16	38.54
2	0.99	33.11
3	0.85	28.35
Variables	Component 1 Loadings (Health)	Relative importance (%)
Number of hospital care visits, 6 months before the decision	0.6985	43
Inpatient care days 6 month before the decision	0.2811	17
Drug doses a day 6 month before the decision	0.6580	40
Labor market attachment		
Component	Eigenvalue	Proportion explained (%)
1	1.17	59
2	0.82	41
Variables	Component 1 Loadings (Attachment)	Relative importance (%)
Employed at the time of decision	0.7071	50
Highest educational level	0.7071	50

Note: Number of observations 11,557

Table A4: Results from the analysis of effects on employment. An individual is being defined as employed if his or her labor income is greater than one price base amount.

	Employment					
	All	Mean [a]	New	Mean [a]	Ongoing	Mean [a]
Year 1	0.31***	0.70	0.17	0.71	0.77***	0.67
	(0.11)		(0.11)		(0.27)	
Year 2	0.20*	0.66	0.12	0.67	0.43	0.62
	(0.11)		(0.11)		(0.26)	
Year 3	0.20*	0.65	0.09	0.67	0.51*	0.59
	(0.11)		(0.11)		(0.27)	

Note: Estimated standard errors are corrected for the estimated instrument and using a robust covariance matrix. [a]: Mean of dependent variable in the control group. The number of observations for each group are as follows: all: 11,557, New: 8,226, Ongoing: 3,331. . *, ** and *** denote statistical significance at the ten, five and one percent level, respectively
